# Acute small fiber neuropathy after Oxford‐AstraZeneca ChAdOx1‐S vaccination: A report of three cases and review of the literature

**DOI:** 10.1111/jns.12509

**Published:** 2022-08-29

**Authors:** Molly G. Abbott, Zahra Allawi, Monika Hofer, Olaf Ansorge, Stefen Brady, Ricardo Fadic, Gustavo Torres, Ravi Knight, Margarita Calvo, David L. H. Bennett, Andreas C. Themistocleous

**Affiliations:** ^1^ Medical Sciences Division University of Oxford Oxford UK; ^2^ Department of Clinical Neurophysiology Oxford University Hospitals NHS Foundation Trust Oxford UK; ^3^ Department of Neuropathology Oxford University Hospitals NHS Foundation Trust Oxford UK; ^4^ Oxford Muscle Service, Department of Neurology Oxford University Hospitals NHS Foundation Trust Oxford UK; ^5^ Department of Neurology Pontificia Universidad Católica de Chile Santiago Chile; ^6^ Physiology Department Pontificia Universidad Católica de Chile Santiago Chile; ^7^ Millennium Nucleus for the Study of Pain (MiNuSPain) Santiago Chile; ^8^ Nuffield Department of Clinical Neurosciences University of Oxford Oxford UK

**Keywords:** COVID‐19 vaccination, neuropathic pain, peripheral neuropathy, small fibre neuropathy

## Abstract

Small fiber neuropathy usually presents with gradual and progressive chronic length‐dependent pain. Acute small fiber neuropathy is rarely reported. Three patients with acute onset neuropathic pain after Oxford‐AstraZeneca ChAdOx1‐S vaccination are described. Two patients were identified at the Oxford University NHS Foundation Trust, Oxford, UK and one patient in Red de Salud UC Christus, Santiago, Chile. All patients underwent a clinical assessment that included a detailed neurological examination, laboratory investigations, nerve conduction studies, thermal threshold testing, and skin biopsy for intra‐epidermal nerve fiber density. Patients seen in Oxford underwent MRI of the brain and spinal cord. Cerebrospinal analysis was not performed. Neuropathic symptoms (burning pain, dysaesthesias) developed in the hands and feet within 2 weeks of vaccination. On clinical examination, there was pinprick and thermal hyposensitivity in the area of neuropathic pain. Laboratory investigation, nerve conduction tests, sympathetic skin responses, and MRI showed no relevant abnormalities. Thermal thresholds were abnormal and intra‐epidermal nerve fiber density in the lower leg was reduced. In two cases symptoms persist after several months. Three cases of definite acute small fiber neuropathy after Oxford‐AstraZeneca ChAdOx1‐S vaccination are described. At follow up, neuropathic pain was present in two of the patients.

## 1 BACKGROUND AND AIMS

Small fiber neuropathy is defined as a structural abnormality of distal termination of the small fibers (which are the thinly myelinated and unmyelinated fibers of sensory afferent and autonomic neurons). Chronic pain, in a length‐dependent pattern, is almost always the presenting complaint with gradual onset and slow progression.^1^ In contrast, acute onset sensory small fiber neuropathy is rare. To date, only several case reports of acute onset small fiber neuropathy are described.^2^, ^3^, ^4^, ^5^, ^6^, ^7^ In these instances, patients present with acute onset numbness and burning pain, either in the extremities or more generalized, that reaches a peak within 6 weeks. Some patients report symptoms after vaccination^3^, ^4^, ^6^, ^7^ or an antecedent illness, such as diarrhea or COVID‐19 infection. Some observers speculate that acute small fiber neuropathy may represent a sensory variant of Guillain‐Barré syndrome, if symptoms peak within 6 weeks. In our case series, we report three patients with acute onset small fiber neuropathy after Oxford‐AstraZeneca ChAdOx1‐S vaccination. Symptoms within two of our three patients still persist after several months.

Two patients were identified at the Oxford University NHS Foundation Trust, Oxford, UK and a single patient in Red de Salud UC Christus, Santiago, Chile. All patients underwent a clinical assessment that included a detailed neurological examination, laboratory investigations, nerve conduction studies, thermal threshold testing, and skin biopsy for intra‐epidermal nerve fiber density. Patients seen in Oxford underwent MRI of the brain and spinal cord. Cerebrospinal analysis was not performed. A comprehensive structured upper and lower limb neurological examination was performed to detect clinical signs of a peripheral neuropathy. The examination included assessment of temperature, light touch and pinprick sensation, joint position proprioception, vibration perception, deep‐tendon reflexes, muscle bulk, and motor power. Orthostatic hypotension, as a marker of autonomic neuropathy, was assessed by measuring lying and standing blood pressure in accordance with established protocols. Orthostatic hypotension was defined as either a 20 mm Hg reduction in systolic or a 10 mm Hg reduction in diastolic blood pressure assessed 2 minutes after standing. Laboratory investigations included: general hematology (full blood count); hematinic (vitamin B12, folate); general biochemistry (urea, creatinine, electrolytes, liver function tests, C‐reactive protein, HbA1c, angiotensin‐converting enzyme, triglycerides, copper); endocrinology (thyroid stimulating hormone, T4); specialist protein (serum protein electrophoresis, immunoglobulins, serum free light chains); autoimmune serology (ANA, ANCA, tissue transglutaminase); and microbiology (HIV, syphilis, hepatitis B and C). Nerve conduction studies were performed in line with those recommended by the American Academy of Neurology and American Association of Electrodiagnostic Medicine. Sensory nerve responses were recorded from the radial, median, ulnar, and sural nerve. Motor nerve responses and F waves were recorded from the median, ulnar, peroneal, and tibial nerves. The minimum case definition criterion for electrodiagnostic confirmation of distal symmetrical polyneuropathy was an abnormality of any attribute of nerve conduction in two separate nerves, one of which was the sural nerve. Sympathetic skin response was tested from the hand and foot. Cold and warm detection thresholds, using methods of limits, were measured from the dorsum of the right foot and compared to age and gender reference ranges. Skin biopsy samples were taken and analyzed in accordance with the consensus document produced by the European Federation of Neurological Societies/Peripheral Nerve Society Guideline on the utilization of skin biopsy samples in the diagnosis of peripheral neuropathies.^8^ Skin biopsies were taken 10 cm proximal to the lateral malleolus with a disposable 3‐mm punch biopsy circular blade. Immunohistochemistry for protein gene product (PGP) 9.5 was performed using brightfield immunohistochemistry (patient 1 and 2) or indirect immunofluorescence (patient 3). Intra‐epidermal nerve fiber density was considered abnormal if it was below the fifth centile for age‐ and sex‐matched healthy controls.^9^, ^10^

All patients signed written consent in accordance with the Declaration of Helsinki. Oxford patients were recruited as part of the Pain in Peripheral Nerve Lesions study (National Research Ethics Service of the United Kingdom, Ref: 18/SC/0263), and Chilean patient was recruited as part of the MINUSPAIN study, which was approved by the Institutional Ethics Committee of the Pontificia Universidad Católica de Chile (project ID: 210112006).

**FIGURE 1:**
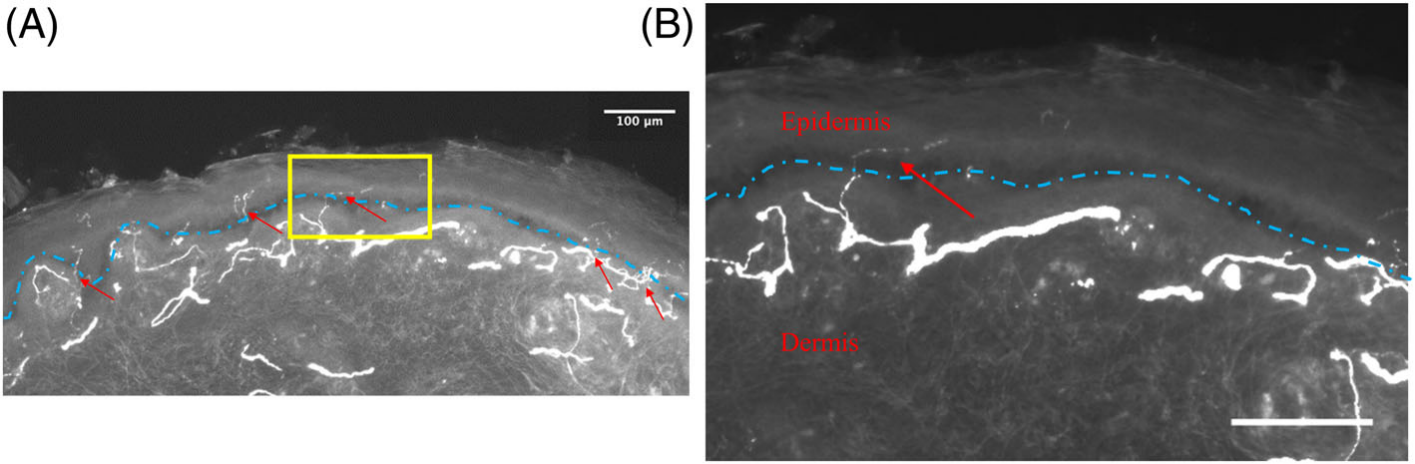
Skin biopsy demonstrating small fiber pathology. (A) Low‐power image of skin biopsy from patient 3, showing protein gene product 9.5‐immunoreactive fibers (arrow heads) crossing the basement membrane of the epidermis (dashed line). The area framed in yellow is shown enlarged in the next image. (B) High‐power image showing single intraepidermal nerve fiber (red arrow) crossing the basement membrane of the epidermis (blue dashed line). Scale bars represent 100 μm.

Patients 1 and 2 were assessed in Oxford, UK, and Patient 3 was seen in Santiago, Chile. Main clinical findings and investigations are summarized in Table 1. Representative images of intra‐epidermal nerve fiber density are shown in Figure 1.

**TABLE 1:** Summary of relevant clinical findings and investigations

	Patient 1	Patient 2	Patient 3
Gender	Female	Female	Male
Onset of symptoms and history	7 days after vaccination. Dysaesthesias and altered temperature sensation on hands and feet extending proximally	10 days after vaccination. Bilateral neuropathic pain and paraesthesias in the hands and feet. Cheeks, nose and tongue numbness and paraesthesias	15 days after vaccination. Dysaesthesias and altered temperature sensation on hands and feet
Neurological examination	Reduced vibration sensation at right toe. No other abnormal findings	Bilateral pinprick hyposensitivity in feet. No other abnormal findings	Bilateral pinprick hyposensitivity up to 15 cm proximal to malleoli
Laboratory tests	ANA 1:80, speckled, IgA mildly elevated, contextually a non‐specific finding	ANCA and ANA positive, but PR3 and MPO titers low (atypical pattern)	No abnormalities
Clinical neurophysiology^a^			
Sural nerve (sensory)			
Amplitude	8.4 μV (≥5 μV)	7.9 μV (≥5 μV)	7.6 μV (≥5 μV)
Conduction velocity	44 m/s (≥39 m/s)	48 m/s (≥39 m/s)	47 m/s (≥39 m/s)
Radial nerve (sensory)			
Amplitude	22.7 μV (≥15 μV)	26.1 μV (≥15 μV)	25.8 μV (ulnar antidromic) (≥15 μV)
Conduction velocity	64 m/s (≥49 m/s)	57 m/s (≥49 m/s)	52 m/s (≥49 m/s)
Peroneal nerve (motor)			
Amplitude	9.4 mV (≥2 mV)	2.5 mV (≥2 mV)	4.8 mV (≥2 mV)
Conduction velocity	47.0 m/s (≥39 m/s)	49.7 m/s (≥39 m/s)	43 m/s (≥39 m/s)
F wave (min latency)	54.5 ms (≥58 ms)	54.3 ms (≥58 ms)	Not done
Median nerve (motor)			
Amplitude	10.0 mV (≥4 mV)	10.7 mV (≥4 mV)	10.1 mV (≥4 mV)
Conduction velocity	54.5 m/s (≥49 m/s)	56.2 m/s (≥49 m/s)	56 m/s (≥49 m/s)
F wave (min latency)	27.8 ms (≥32 ms)	27.0 ms (≥32 ms)	Not done
Sympathetic skin response	Present	Present	Present
Electromyography	Normal	Normal	Chronic bilateral L5, left C6 radiculopathies
Thermal thresholds (mean ± SD)			
Warm detection^b^	45.4 ± 0.4°C (45°C)	38.0 ± 1.9°C (44°C)	45.2 ± 2.2°C (45°C)
Cold detection^b^	24.9 ± 0.5°C (27°C)	30.1 ± 0.2°C (27°C)	26.2 ± 1.1°C (27°C)
MRI head and spine	Multiple perineural cysts attached to bilateral L5‐S2 nerve roots deemed incidental. Cystic dilatation of the distal spinal cord central canal, no change from previous imaging	Prominent central canal within the spinal cord from C4/C5 to mid T1 vertebral level	Not performed
Intraepidermal nerve fiber density	3.0 fibers/mm	3.4 fibers/mm	8.0 fibers/mm
0.05 quantile (median) for age and gender	3.2 (7.6) fibers/mm	4.3 (9.8) fibers/mm	8.7 (9.3) fibers/mm

^a^ Reference range included in parentheses for each neurophysiological measure.

^b^ Age and gender warm and cold detection thresholds are included in parentheses for each patient.

## 2 CASE REPORTS

Patient 1, a patient in her late 60s, developed symptoms 7 days after the first Oxford‐AstraZeneca ChAdOx1‐S vaccine (February 18, 2021), and presented to health care staff 7 weeks after symptom onset. The main symptoms were dysaesthesias and altered temperature sensation on the hands and feet extending to the elbows and knees. There was a remote history of spinal cord syrinx in the lumbar region with repeat MRI imaging showing no change. On clinical examination, there was mild reduction in vibration sensation at the right toe, with normal pinprick sensation. Thermal detection thresholds and intra‐epidermal nerve fiber density were abnormal in the area of neuropathic pain confirming a diagnosis of small fiber neuropathy. Laboratory investigations identified a low positive ANA and mildly elevated IgA, not considered clinically relevant. Nerve conduction studies, EMG, and sympathetic skin response were normal. MRI of the brain and spinal cord identified incidental perineural cysts and cystic dilatation of the distal spinal cord central canal with no evidence of a syrinx. Within 3 months neuropathic pain symptoms had resolved. After second dose of the AstraZeneca COVID‐19 vaccine symptoms returned (June 17, 2021), only to once again settle after 3 months. At follow up, 9 months after symptom onset, the patient was symptom‐free, and neurological examination was normal. Patient received a third dose on December 13, 2021.

Patient 2, a patient in her mid‐50s, developed neuropathic symptoms 10 days after the first Oxford‐AstraZeneca ChAdOx1‐S vaccine (March 13, 2021), and presented to health care staff at the time of symptom onset. The main symptoms were bilateral burning pain, dysaesthesias, and altered temperature sensation on the hands and feet extending proximally. Intra‐epidermal nerve fiber density was abnormal in the area of neuropathic pain confirming a diagnosis of small fiber neuropathy. Nerve conduction studies, EMG, sympathetic skin response, and thermal detection thresholds were normal. MRI of the brain and spinal cord identified dilatation of the cervical spinal cord central canal with no evidence of a syrinx. Laboratory tests identified positive ANCA and ANA that were not clinically relevant as PR3 and MPO titers were low. Due to the severity of pain and intrusive symptoms, gabapentin and amitriptyline were started. Neuropathic symptoms worsened after the administration of the second AstraZeneca COVID‐19 vaccine (May 27, 2021). In October 2021 patient was reviewed by pain management center who changed gabapentin to pregabalin due to cognitive side effects and provided guidance on pathways for psychological support.

Although autoantibodies were detected in patient 1 and 2, these were at low levels that can be seen in healthy individuals. Furthermore, the patients did not show features to suggest a concomitant autoimmune process such as Systemic Lupus Erythematosus, Sjogren's syndrome, or small vessel vasculitis.

Patient 3, a patient in his early 60s, developed symptoms 15 days after the first Oxford‐AstraZeneca ChAdOx1‐S vaccine (August 20, 2021), and presented to health care staff 8 weeks after symptom onset. The main symptoms were dysaesthesias and altered temperature sensation on hands and feet. Clinical examination showed bilateral pinprick hyposensitivity 15 cm proximal to malleoli. Thermal detection thresholds and intra‐epidermal nerve fiber density were abnormal in the area of neuropathic pain, confirming a diagnosis of small fiber neuropathy. Full blood count, biochemical profile, serum and urine immunofixation, vitamin B12 and B6 levels were normal. Extractable nuclear antigen panel, rheumatoid factor, C‐reactive protein, and ESR were also normal. Nerve conduction studies and sympathetic skin response were normal. EMG study showed bilateral L5 and left C6 radiculopathies, that were chronic, without active denervation, and present in older studies. A whole body FDG‐PET/CT was negative for a primary malignancy. Symptoms have not resolved and the patient refused a second dose of the Oxford‐AstraZeneca ChAdOx1‐S vaccine.

## 3 INTERPRETATION

We report a case series of acute small fiber neuropathy after Oxford‐AstraZeneca ChAdOx1‐S vaccination. In all the cases patients satisfied criteria for definite small fiber neuropathy. The criteria include clinical evidence of small fiber neuropathy (neuropathic symptoms in the distal extremities and evidence for pinprick or thermal hyposensitivity in the area of neuropathic pain), normal nerve conduction studies, and reduced intra‐epidermal nerve fiber density.^1^ Of concern is two patients were still experiencing chronic neuropathic pain at follow‐up. The development of our patients' presentation soon after vaccination and exclusion of other known etiologies, raises the possibility of an association. However, as this satisfies only a minority of the nine Bradford Hill criteria (which should be satisfied to demonstrate a causal relationship), we would not assign a causal relationship between Oxford‐AstraZeneca ChAdOx1‐S vaccination and acute small fiber neuropathy. A previous case of small fiber neuropathy‐ganglionopathy is described after the Oxford‐AstraZeneca ChAdOx1‐S vaccination.^4^ In this case, the patient reported painful cold sensation that progressed over 2 weeks. Clinical examination revealed cold hypoesthesia of the legs, normal nerve conduction studies, and reduced distal leg intraepidermal nerve fiber density, and quantitative sudomotor axon reflex was abnormal in the right forearm.

Although the literature is limited, acute small fiber neuropathy presents after vaccination, antecedent illness or with no clear precipitant. Acute small fiber neuropathy is reported after a variety of vaccinations that include Pfizer/BioNTech COVID‐19 vaccine,^7^ human papillomavirus vaccine,^3^ and vaccinations against varicella, rabies, and Lyme disease.^6^ Similar to our cases, symptom onset is within 2 weeks after vaccination and presents in a length‐dependent pattern. The only exception was the human papillomavirus vaccine, where there was a more generalized and non‐length‐dependent pattern. Antecedent illnesses include urinary tract infection, diarrhea^5^ or primary COVID‐19 infection.^11^ In these cases, the onset is more often between 2 and 6 weeks post‐exposure. Furthermore, small fiber neuropathy was detected in 62.5% (10/16) patients diagnosed with post‐acute sequelae of SARS CoV‐2 infection.^12^ Other neurological complications are linked to COVID‐19 vaccination.^13^ The most commonly reported is facial nerve palsy. There is a small increased risk of hospital admission from Guillain‐Barré syndrome, myasthenic disorder, and Bell's palsy after Oxford‐AstraZeneca ChAdOx1‐S vaccination; however, this risk is still lower than after primary SARS‐CoV‐2 infection.^13^

We can only speculate as to possible pathophysiological mechanisms of acute small fiber neuropathy. Reports implicate immune or inflammatory mechanisms. Several cases of small fiber neuropathy after preceding illness showed albumin‐cytological dissociation on cerebrospinal fluid analysis.^5^ This suggests proximal demyelination normally observed in inflammatory demyelinating polyradiculoneuropathies. Although in a separate case series there was a dramatic improvement after corticosteroid therapy.^2^ We propose that acute small fiber neuropathy may be caused by the combination of an underlying, unrecognized risk factor with another trigger, such as an immune response to an infection or vaccination.

In conclusion, we report three cases of acute small fiber neuropathy after Oxford‐AstraZeneca ChAdOx1‐S vaccination, with two patients developing chronic pain. Hence, we hypothesize that acute small fiber neuropathy may develop in the context of an unknown underlying risk factor combined with an immune or inflammatory trigger. Our case series highlights that acute small fiber neuropathy can follow COVID‐19 vaccination and lead to chronic complications.

## AUTHOR CONTRIBUTIONS

*Conception and design of the study*: Andreas C. Themistocleous and Molly G. Abbott. *Acquisition and analysis of data*: all authors. *Drafting a significant portion of the manuscript or figures*: all authors.

## CONFLICT OF INTEREST

The authors declare no potential conflict of interest.

## Data Availability

The data that support the findings of this study are available on request from the corresponding author.
